# Clinical operations of academic versus non-academic emergency departments: a descriptive comparison of two large emergency department operations surveys

**DOI:** 10.1186/s12873-019-0285-7

**Published:** 2019-11-21

**Authors:** Martin A. Reznek, Sean S. Michael, Cathi A. Harbertson, James J. Scheulen, James J. Augustine

**Affiliations:** 1 Department of Emergency Medicine, University of Massachusetts Medical School Worcester Massachusetts 55 Lake Avenue North, Worcester, MA 01655 USA; 2 Department of Emergency Medicine, University of Colorado School of Medicine, Aurora, CO USA; 3 Department of Emergency Medicine, the Johns Hopkins University School of Medicine, Baltimore, MD USA; 4 Department of Emergency Medicine, Wright State University Boonshoft School of Medicin Dayton Ohio USA and US Acute Care Solutions, Canton, OH USA

**Keywords:** Emergency department, Operations, Academic, Community, Benchmarking

## Abstract

**Background:**

Academic and non-academic emergency departments (EDs) are regularly compared in clinical operations benchmarking despite suggestion that the two groups may differ in their clinical operations characteristics. and outcomes. We sought to describe and compare clinical operations characteristics of academic versus non-academic EDs.

**Methods:**

We performed a descriptive, comparative analysis of academic and non-academic adult and general EDs with 40,000+ annual encounters, using the Academy of Academic Administrators of Emergency Medicine (AAAEM)/Association of Academic Chairs of Emergency Medicine (AACEM) and Emergency Department Benchmarking Alliance (EDBA) survey results. We defined academic EDs as primary teaching sites for emergency medicine (EM) residencies and non-academic EDs as sites with minimal resident involvement. We constructed the academic and non-academic cohorts from the AAAEM/AACEM and EDBA surveys, respectively, and analyzed metrics common to both surveys.

**Results:**

Eighty and 454 EDs met inclusion criteria for academic and non-academic EDs, respectively. Academic EDs had more median annual patient encounters (73,001 vs 54,393), lower median proportion of pediatric patients (6.3% vs 14.5%), higher median proportion of EMS patients (27% vs 19%), and were more commonly designated as Level I or II Trauma Centers (94% vs 24%). Median patient arrival-to-provider times did not differ (26 vs 25 min). Median length-of-stay was longer (277 vs 190 min) for academic EDs, and left-before-treatment-complete was higher (5.7% vs 2.9%). MRI utilization was higher for academic EDs (2.2% patients with at least one MRI vs 1.0 MRIs performed per 100 patients). Patients-per-hour of provider coverage was lower for academic EDs with and without consideration for advanced practice providers and residents.

**Conclusions:**

Demographic and operational performance measures differ between academic and non-academic EDs, suggesting that the two groups may be inappropriate operational performance comparators. Causes for the differences remain unclear but the differences appear not to be attributed solely to the academic mission.

## Background

In 2017, investigators reported the clinical, educational, and research contributions of academic emergency departments (EDs) in the Unites States (US) based on the results of the Academy of Academic Administrators of Emergency Medicine (AAAEM)/Association of Academic Chairs of Emergency Medicine (AACEM) benchmarking survey [1]. While not the focus of that investigation, the findings suggested that academic and non-academic EDs may differ in their clinical operations characteristics and outcomes. To date, this potential difference between academic and non-academic EDs appears to be relatively unacknowledged by healthcare oversight entities. In the US for example, the Centers for Medicare and Medicaid Services, the dominant national healthcare insurer, and the Joint Commission, the dominant hospital accrediting body, do not separate or distinguish academic versus non-academic EDs in their reporting of ED clinical performance measures [2, 3]. In addition, leaders of both academic and non-academic EDs anecdotally report their parent organizations having compared their EDs’ performances to benchmarking datasets that include, or are even dominated by, the other type of ED. If the two types of EDs in fact do differ in their characteristics, these current benchmarking practices would be suboptimal because relevant comparator selection is essential for meaningful benchmarking [4].

The AAAEM/AACEM survey did not allow for internal, direct comparison between pure academic and non-academic EDs, so it remained unclear if the characteristics and outcomes for the two were truly different. Other national benchmarking efforts related to ED clinical operations also exist in the US, but they too do not allow for such an internal, direct comparison. The Medical Group Management Association administers an ED survey, but it has limited clinical operations data and includes few academic programs [1, 5]. The National Hospital Ambulatory Medical Care Survey, designed to characterize ED visits at the population level, is limited in clinical operations metrics, and is limited in its methodology of distinguishing academic versus non-academic departments [6]. The Emergency Department Benchmarking Alliance (EDBA) administers an annual survey focused on ED performance measures to a large number of “community” EDs and “academic” EDs. However “academic” is broadly defined as any participation in resident training, regardless of residency type or daily resident staffing levels [7].

Since no single ED data comparison effort exists to compare appropriately the clinical operations of academic versus non-academic EDs, we sought to investigate differences between these two groups by combining data from the AAAEM/AACEM and EDBA performance surveys. Both sources are characterized by high response rates, include comprehensive clinical operations metrics, have significant overlap in their definition of clinical metrics [8], and their methodologies allow for the accurate establishment of academic and non-academic cohorts, repsectively [1, 8].

## Methods

### Study setting and population

We performed a descriptive, comparative analysis of the most current AAAEM/AACEM and EDBA ED survey results for adult-only and general EDs in the US with 40,000+ annual encounters. We excluded pediatric-only EDs and freestanding EDs because of different clinical operations outcomes and work environments (AAAEM/AACEM and EDBA report data from these EDs separately). To optimize balanced comparisons, we excluded EDs with fewer than 40,000 annual patient encounters because only one academic ED was in that volume stratum. The Johns Hopkins Medicine Institutional Review Board approved the investigation as exempt.

### Survey Descriptions

The AAAEM/AACEM survey is administered annually to allopathic, academic emergency medicine (EM) programs with one of the following inclusion criteria: (1) full department in a Liaison Committee on Medical Education (LCME) accredited medical school, (2) division or section of another department in an LCME medical school that hosts an Accreditation Council for Graduate Medical Education (ACGME) accredited EM residency program, or (3) hospital-based department, division, or section affiliated with an LCME medical school and hosts an ACGME-accredited EM residency program [9]. Survey development details are available in previous publication [1]. Within that survey instrument (Additional file 1: Table S1), programs reported clinical operations metrics for one or more EDs with which they were affiliated. Programs reported each ED as being a primary academic site, an academic affiliate site, a community site, or a freestanding ED. We analyzed the AAAEM/AACEM survey results for the 2016-17 academic year (July 1, 2016 - June 30, 2017).

The EDBA survey is administered annually to EDBA member institutions and former member institutions that submitted survey responses in prior years. EDBA membership and survey development details are available in previous publications and the EDBA website [7, 8, 10, 11]. Within the survey instrument (Additional file 2: Table S2), respondents reported clinical operations metrics attributable to the ED site level, labeling each ED as academic-affiliated (defined as participating in training EM and other residents) or not academic-affiliated. We analyzed the EDBA survey results for the 2017 calendar year.

Both surveys were voluntary, but respondents were afforded the incentive of receiving de-identified results and analyses of the surveys. The EDBA dataset available to the authors for analysis was de-identified (although hospital zip code was available), and the AAAEM/AACEM dataset was not de-identified (necessary for academic site validation, described below).

We excluded EDs that did not provide their annual volume or admission rate (or sufficient information to calculate either) and those missing all other clinical operations data.

### Primary comparison

Our primary comparison was of operationally academic EDs (defined as a primary teaching site for an EM residency) to non-academic EDs (EDs without substantial resident involvement in routine operations).

We constructed the academic cohort beginning with adult and general EDs from the AAAEM/AACEM survey who self-identified as a “primary academic” site. This designation ensured that the cohort included only primary teaching sites for ACGME-accredited residencies and excluded non-primary teaching sites and community sites that may have been otherwise affiliated with an academic department of EM. We verified that these EDs reported greater than 24 hours of resident coverage per day. Among the subset reporting fewer than 24 hours (presumed to be an erroneous entry) or with missing resident coverage data (n=15), we searched the program’s website or contacted the program to verify that each program met criteria as a primary teaching site. We excluded remaining EDs with fewer than 40,000 annual patient encounters.

We constructed the non-academic cohort beginning with all EDs from the EDBA survey who self-reported as not being “academic”. Since academic status was not clearly defined in the EDBA survey tool, EDs responding affirmatively to “academic” status included both primary and non-primary academic EDs otherwise affiliated with an academic program. Consequently, we may have excluded sites with minimal academic mission expectations (eg. one EM resident per day), but this exclusion was necessary to maximize specificity for our non-academic cohort. Following a similar specificity strategy, we excluded any site not identifying as “academic” but still reporting more than 12 hours of resident coverage per day. We excluded remaining EDs with less than 40,000 annual patient encounters and pediatric EDs.

### Outcomes

We analyzed all operational metrics for which both surveys used a common definition. Most metrics were available directly from survey respondent entries, but some metrics (eg. admission rate) were calculated from other directly-entered values. Details of metric definitions and calculation formulae are available as supplemental material accompanying the online article.

Left Before Treatment Complete (LBTC) was reported in the EDBA but not in the AAAEM/AACEM results. AAAEM/AACEM instead reported the sub-categories of LBTC including: patients leaving prior to, or following, a medical screening exam and leaving against medical advice/eloping. We added these subcategories together to calculate LBTC for AAAEM/AACEM survey respondents.

We chose also to include resource utilization metrics which were queried differently in the two surveys. EDBA queried for the number of individual procedures with a modality category (“x-rays”, “CT scans”, “MRI images”, and “ultrasounds”) performed per 100 patient encounters, and AAAEM/AACEM queried for number of patients that received one or more of each imaging modalities per encounter (which we divided by total patient encounters), raising the possibility of AAAEM/AACEM reporting lower resource utilization when compared to EDBA. We included these metrics, despite the potential for AAAEM/AACEM under-reporting compared to EDBA, because a finding of higher resource utilization among AAAEM/AACEM respondents, compared to EDBA, would be meaningful.

### Data analysis

Our analytic paradigm was that different patient populations, processes, staffing models, and implicit priorities may drive between-group variation between academic EDs, compared to non-academic EDs, more so than within-group variation. Thus, we calculated descriptive statistics and reported 95% two-sided confidence intervals, rather than hypothesis tests, because they could assess both practical and statistical significance. We avoided distributional assumptions and calculated confidence intervals for medians and interquartile ranges (IQRs) using smoothed empirical likelihood quantile estimates based on a kernel density function [12]. For metrics expected to vary with annual ED census, we also stratified based on volume bands used by prior authors (40k-59k, 60k-79k, 80k-99k and ≥100k) [13]. We used SAS 9.4 and JMP Pro 14 (SAS Institute Inc., Cary, NC) for analyses.

### Secondary analysis

To maximize balanced comparisons, we performed a secondary analysis in which we used Ward’s agglomerative hierarchical clustering to create a 1:1 matched sample set on volume, ED admission rate, trauma designation, and region. Because our primary (unmatched) dataset had a greater-than 5:1 ratio of non-academic to academic EDs, we selected multiple clustering variables to maximize similarity of matched sites over a number of dimensions. Clustering only on volume risked creating a secondary dataset that was equally heterogenous, only smaller. Thus our secondary (matched) analysis included 80 unique non-academic EDs to compare with the 80 academic EDs using the same approach as the primary analysis.

## Results

Among 108 US academic departments of EM sent the AAAEM/AACEM survey, 75 (69.4%) responded. All but one reported data for at least one primary academic clinical site, and five reported data for multiple primary academic sites. Among approximately 2,000 individual EDs sent the EDBA survey, 1,717 (~85.9%) responded.

From the 84 primary academic adult and general EDs reported in the AAAEM/AACEM dataset, we excluded one ED with fewer than 40,000 annual visits and three EDs missing all operational data. (Pediatric EDs were reported in a separate database by the AAAEM/AACEM surveyors and therefore pre-excluded). This resulted in a total of 80 academic EDs.

**Fig. 1:**
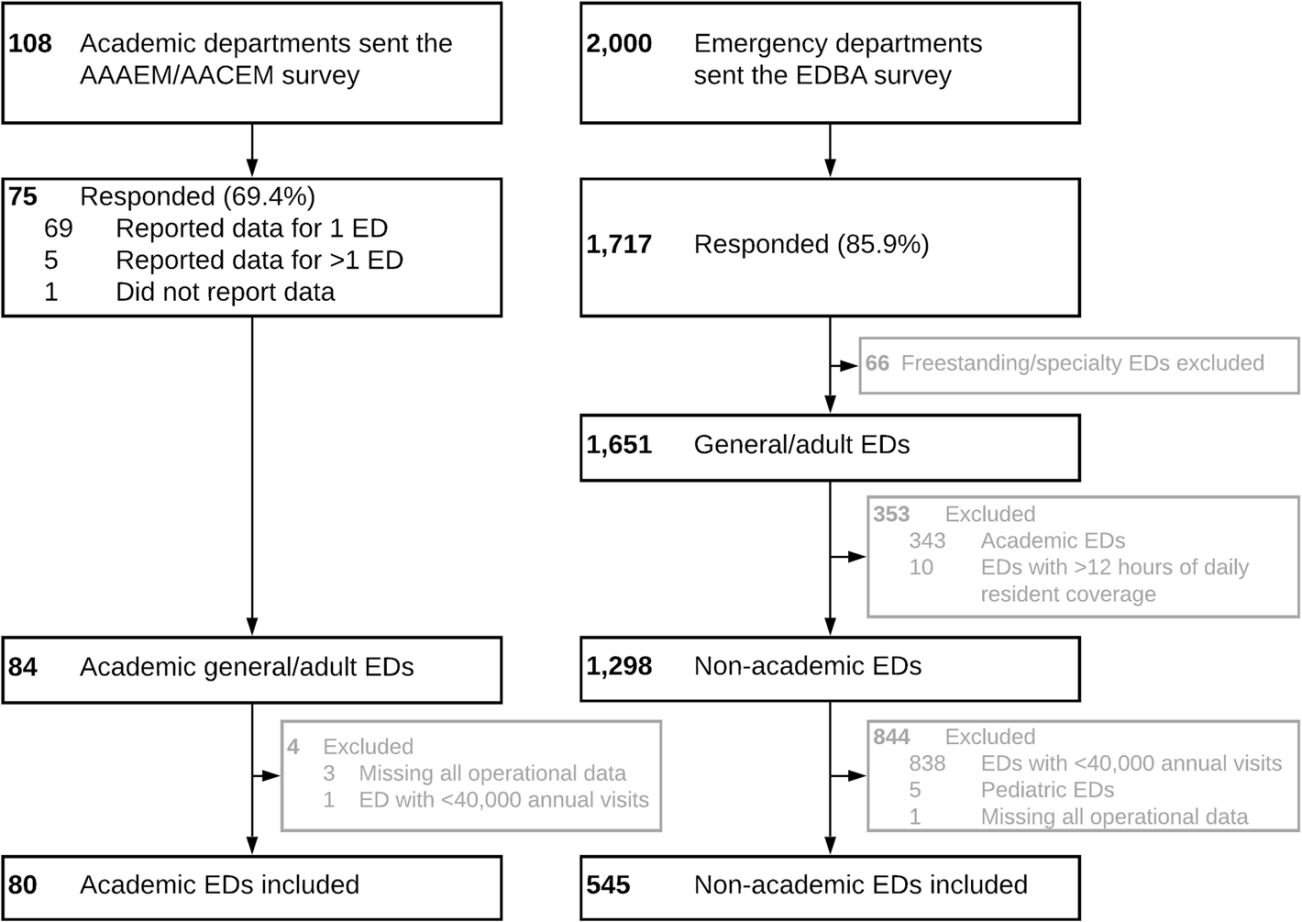
Study flow

From the 1,651 general service US EDs in the EDBA dataset, which pre-excluded freestanding and “specialty” EDs, we excluded 343 EDs that self-identified as academic and 10 others reporting greater than 12 hours of daily resident coverage. From this non-academic subset (n=1298), we excluded 838 EDs with fewer than 40,000 annual visits. We excluded 1 ED missing all operational data and 5 remaining pediatric EDs. This resulted in a total of 454 non-academic EDs. The study flow is shown in Figure 1.

Patient volume, geographic region of the US and the American College of Surgeons trauma center level designation of the included EDs are shown in Table 1.

**Table 1:** ED Patient Volume, Geographic Location and Trauma Center Designation

	Academic EDs (*n* = 80)	Non-Academic EDs (*n* = 454)	Matched Non-Academic EDs (*n* = 80)
Volume strata			
40 k–59 k	20 (25%)	295 (65%)	29 (36%)
60 k–79 k	32 (40%)	118 (26%)	30 (38%)
80 k–99 k	17 (21%)	32 (7%)	14 (18%)
≥100 k	11 (14%)	9 (2%)	7 (8.8%)
Region			
Central	17 (21%)	66 (15%)	19 (24%)
Northeast	27 (34%)	68 (15%)	16 (20%)
South	20 (25%)	241 (53%)	24 (30%)
West	16 (20%)	77 (17%)	21 (26%)
Missing	0	2 (< 1%)	0
Trauma center designation			
I or II	75 (94%)	91 (20%)	63 (79%)
III or IV	0	101 (22%)	4 (5.0%)
Not designated	5 (6%)	262 (58%)	13 (16%)

*ED* Emergency department

**Fig. 2:**
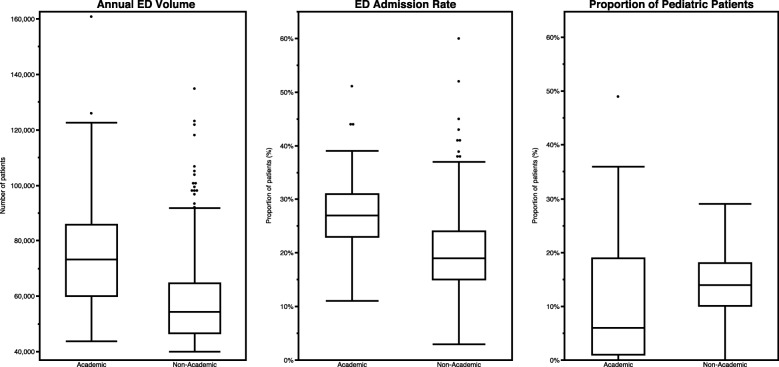
Annual patient volume, admission rate and proportion of pediatric patients

Tables 2 and 3 show measures for which academic and non-academic EDs did and did not differ in the primary analysis, respectively, with Fig. 2 highlighting key differences graphically.

**Table 2:** Characteristics for which academic and non-academic EDs differed in the primary analysis

	Academic EDs (*n* = 80)	Non-Academic EDs (*n* = 454)	Matched Non-Academic EDs (n = 80)
Demographic and operational characteristics			
Annual ED census volume			
Median (95% CI)	73,011 (67,605 to 77,799)	54,393 (52,663 to 56,140)	65,378 (61,315 to 70,855)
IQR (95% CI)	60,088 (56,150 to 64,597) – 86,125 (80,633 to 95,482)	46,678 (45,651 to 47,777) – 64,889 (62,726 to 67,369)	54,069 (49,288 to 58,397) – 82,987 (74,320 to 91,372)
Pediatric mix (%)			
Median (95% CI)	6.3 (3.6 to 13.7)	14.5 (13.8 to 15.2)	14.1 (12.7 to 15.9)
IQR (95% CI)	1.3 (0.4 to 2.5) – 19.3 (15.3 to 26.1)	10.4 (9.3 to 11.5) – 18.2 (17.5 to 18.9)	10.0 (7.4 to 12.0) – 18.4 (16.8 to 20.2)
ED admission rate (%)			
Median (95% CI)	27 (25 to 28)	19 (18 to 20)	24 (23 to 26)
IQR (95% CI)	23 (21 to 25) – 31 (29 to 33)	15 (14 to 16) – 24 (23 to 25)	20 (18 to 22) – 30 (27 to 32)
Non-responders	0	11 (2.4%)	0
Proportion of hospital patients admitted via the ED (%)			
Median (95% CI)	59 (53 to 63)	71 (70 to 72)	68 (63 to 72)
IQR (95% CI)	47 (40 to 51) – 69 (65 to 76)	64 (62 to 66) – 76 (75 to 77)	58 (51 to 62) – 75 (72 to 77)
Non-responders	18 (23%)	224 (49%)	30 (38%)
Proportion of ED patients arriving via EMS (%)			
Median (95% CI)	27 (24 to 29)	19 (18 to 19)	23 (21 to 25)
IQR (95% CI)	20 (17 to 23) – 34 (30 to 37)	15 (14 to 15) – 23 (22 to 24)	19 (17 to 20) – 27 (26 to 30)
Non-responders	15 (19%)	131 (29%)	14 (18%)
Timeliness of care measures			
Left before treatment completion (%)			
Median (95% CI)	5.7 (4.8 to 6.7)	2.9 (2.9 to 3.0)	2.8 (2.2 to 3.1)
IQR (95% CI)	3.5 (2.9 to 4.3) – 8.5 (7.3 to 9.5)	2.0 (1.9 to 2.0) – 4.0 (3.9 to 4.1)	1.8 (1.4 to 2.0) – 4.2 (3.3 to 5.0)
Non-responders	4 (5.0%)	21 (4.6%)	1 (1.3%)
Median LOS (min)			
Median (95% CI)	277 (265 to 293)	190 (184 to 195)	200 (190 to 212)
IQR (95% CI)	246 (229 to 258) – 335 (304 to 357)	162 (157 to 168) – 222 (217 to 229)	174 (164 to 184) – 238 (220 to 253)
Non-responders	6 (7.5%)	20 (4.4%)	0
Median LOS for discharged patients (min)			
Median (95% CI)	241 (230 to 251)	161 (159 to 165)	168 (160 to 177)
IQR (95% CI)	206 (189 to 223) – 276 (258 to 294)	138 (134 to 143) – 190 (185 to 195)	147 (139 to 155) – 197 (182 to 212)
Non-responders	5 (6.3%)	20 (4.4%)	0
Median LOS for admitted patients (min)			
Median (95% CI)	435 (393 to 476)	309 (299 to 321)	316 (297 to 335)
IQR (95% CI)	354 (327 to 378) – 529 (494 to 565)	262 (256 to 269) – 367 (358 to 378)	274 (256 to 289) – 361 (343 to 386)
Non-responders	6 (7.5%)	21 (4.6%)	1 (1.3%)
Median boarding time (min)			
Median (95% CI)	163 (151 to 177)	108 (101 to 116)	120 (102 to 145)
IQR (95% CI)	126 (107 to 144) – 214 (185 to 252)	79 (73 to 84) – 153 (141 to 165)	77 (60 to 96) – 177 (151 to 202)
Non-responders	11 (14%)	164 (36%)	18 (23%)
Imaging utilization measures			
MR imaging	% of patients with at least one MR	Total MRs per 100 patients	Total MRs per 100 patients
Median (95% CI)	2.2 (1.7 to 2.8)	1.0 (1.0 to 1.1)	1.4 (1.1 to 1.9)
IQR (95% CI)	1.1 (0.6 to 1.7) – 3.4 (2.8 to 4.4)	0.5 (0.5 to 0.6) – 2.1 (1.9 to 2.5)	0.8 (0.6 to 1.0) – 2.8 (2.2 to 3.4)
Non-responders	35 (44%)	124 (27%)	14 (18%)

*ED* Emergency department, *EMS* Emergency medical services, *LOS* Length of stay, *MR* Magnetic resonance, *CI* Confidence interval, *IQR* Interquartile range

**Table 3:** Characteristics for which academic and non-academic EDs did not differ in the primary analysis

	Academic EDs (*n* = 80)	Non-Academic EDs (*n* = 454)	Matched Non-Academic EDs (n = 80)
Demographic and operational characteristics			
Admission rate for patients arriving via EMS (%)			
Median (95% CI)	44 (41 to 48)	42 (41 to 44)	48 (46 to 50)
IQR (95% CI)	37 (29 to 40) – 52 (48 to 59)	36 (35 to 37) – 48 (47 to 50)	42 (37 to 45) – 51 (51 to 58)
Non-responders	27 (34%)	149 (33%)	15 (19%)
Proportion of patients with high CPT code (%)			
Median (95% CI)	75 (71 to 78)	72 (71 to 73)	74 (72 to 76)
IQR (95% CI)	67 (64 to 69) – 81 (79 to 83)	66 (65 to 68) – 77 (76 to 79)	67 (62 to 71) – 80 (77 to 82)
Non-responders	6 (7.5%)	31 (6.8%)	3 (3.8%)
Timeliness of care measures			
Arrival-to-provider time (min)			
Median (95% CI)	26 (23 to 32)	25 (23 to 27)	28 (23 to 32)
IQR (95% CI)	19 (17 to 21) – 42 (34 to 53)	16 (14 to 17) – 37 (34 to 40)	16 (13 to 20) – 39 (34 to 46)
Non-responders	7 (8.8%)	37 (8.1%)	4 (5.0%)
Imaging utilization measures			
X-rays	% of patients with at least one X-ray	Total X-rays per 100 patients	Total X-rays per 100 patients
Median (95% CI)	39 (38 to 41)	42 (41 to 44)	48 (44 to 50)
IQR (95% CI)	36 (32 to 38) – 44 (41 to 48)	35 (33 to 37) – 50 (49 to 52)	40 (34 to 43) – 54 (51 to 59)
Non-responders	37 (46%)	118 (26%)	17 (21%)
CT imaging	% of patients with at least one CT	Total CTs per 100 patients	Total CTs per 100 patients
Median (95% CI)	22 (20 to 25)	23 (21 to 24)	26 (24 to 29)
IQR (95% CI)	17 (15 to 20) – 27 (25 to 29)	16 (14 to 18) – 29 (28 to 31)	21 (18 to 23) – 33 (30 to 38)
Non-responders	33 (41%)	117 (26%)	13 (16%)
US imaging	% of patients with at least one US	Total US per 100 patients	Total US per 100 patients
Median (95% CI)	6.1 (4.4 to 7.7)	6.7 (6.2 to 7.1)	6.3 (5.5 to 7.5)
IQR (95% CI)	2.8 (1.6 to 4.7) – 9.5 (7.5 to 13)	4.9 (4.4 to 5.2) – 9.1 (8.7 to 9.7)	4.5 (3.3 to 5.3) – 8.9 (7.5 to 10.1)
Non-responders	42 (53%)	194 (43%)	29 (36%)

*EMS* Emergency medical services, *CPT* Current Procedural Terminology (a registered trademark of the American Medical Association), *CT* Computerized tomography, *US* Ultrasound, *CI* Confidence interval, *IQR* Interquartile range

Proportion of patients with high CPT code is the proportion of patients with CPT code 99284, 99285 or 99,291

### Patient acuity

Our comparative findings between academic and non-academic EDs related to ED admission rate, proportion of ED patients arriving via EMS, admission rate for patients arriving via EMS, and proportion of patients with high-acuity professional billing codes (Current Procedural Terminology (CPT) codes: 99284, 99285 or 99291) [14] persisted even within volume strata and trauma designation.

The difference in the proportion of hospital patients admitted via the ED among academic versus non-academic EDs was accounted for primarily by lower-volume academic EDs. In the 40k-59k stratum, median 60% (95% CI 50 to 67) of patients in academic centers were admitted via the ED, compared to 71% (95% CI 70 to 73) in non-academic centers. In the 60k-79k stratum, median values were 51% (95% CI 45 to 57) in academic centers and 70% (95% CI 67 to 73) in non-academic centers. In the 80k-99k and ≥100k strata, median values were similar: 66% (95% CI 59 to 71) in academic centers and 70% (95% CI 66 to 74) in non-academic centers.

### Imaging resource utilization

Magnetic Resonance Imaging (MRI) utilization appeared higher among academic centers than non-academic centers (median 2.2 [95% CI 1.7 to 2.8] encounters with MRI(s) per 100 total academic encounters, versus 1.0 [95% CI 1.0 to 1.1] MRIs per 100 non-academic encounters). This difference extinguished in the matched sample analysis, and there did not appear to be a difference between the 42 academic and 76 non-academic level I and II trauma centers who reported this metric (median 2.2 [95% CI 1.6 to 2.8] versus 1.5 [95% CI 1.1 to 2.2], respectively). In general, there was no difference in the median utilization of other imaging modalities, including by volume strata or trauma designation.

### Throughput and flow

**Fig. 3:**
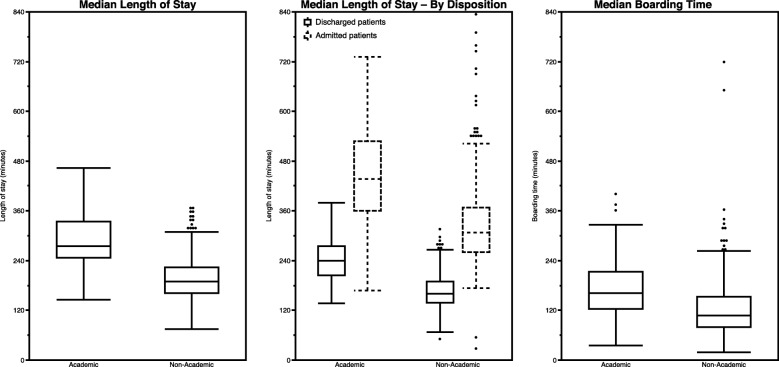
Length of stay and boarding

There was no difference in arrival-to-provider time between academic and non-academic EDs, regardless of volume stratum or trauma designation. The proportion of patients who left before treatment completion (LBTC) was higher and the median length of stay (LOS) was longer among academic EDs, differences which generally persisted across volume strata, regions, and trauma designations. The fastest quartile of academic EDs, in terms of overall LOS, were still slower than the slowest quartile of non-academic EDs. A few outlier academic EDs in the West skewed toward lower LOS. Otherwise, there were few regional LOS differences. In general, median boarding time was longer in academic EDs, most prominently in the 60k-79k and 80k-99k volume strata. While boarding times did typically increase with ED volume, there was no difference in boarding times between academic and non-academic EDs in the 40k-59k and ≥100k strata. Key flow differences are highlighted in Figure 3.

### ED physical factors

Average ED care space size varied from 193 to 1348 square feet, although the median value for academic and non-academic EDs was similar (523 [95% CI 474 to 576] versus 517 [95% CI 488 to 548]). Average care space size tended to be smaller among non-academic EDs in the West and academic EDs in the Northeast. Median annual ED visits per care space was lower for academic EDs (1,170 [95% CI 1,115 to 1,234]) versus non-academic EDs (1,515 [95% CI 1,460 to 1,566]).

### ED staffing

Of 76 academic EDs who reported staffing data, 39 (51%) reported using scribes, compared to 125 of 332 (38%) non-academic EDs. Scribe use increased with ED volume, with 17 of 26 (65%) academic EDs with ≥80,000 annual visits using scribes, compared to 16 of 30 (53%) non-academic EDs.

**Fig. 4:**
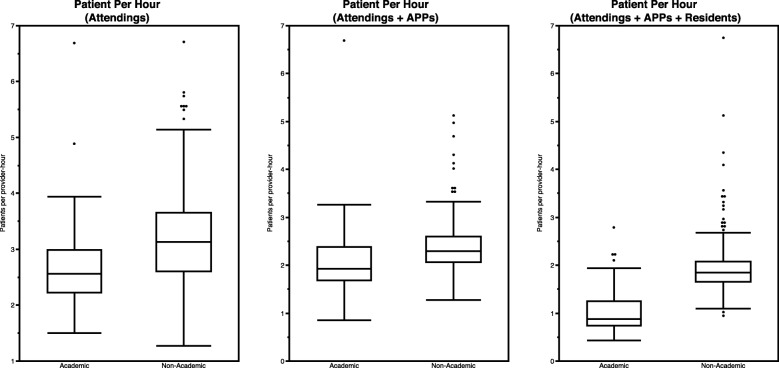
Patients per provider hour

The ratio of daily advanced practice provider (APP) hours to attending physician hours was similar between academic and non-academic EDs (median 0.61 [95% CI 0.51 to 0.73] versus 0.70 [95% CI 0.65 to 0.74]). However, daily patients per provider-hour (PPH) (when stratified by attending hours, attending plus APP hours and attending plus APP and resident hours) differed among academic and non-academic EDs (Table 4 and Figure 4).

**Table 4:** Patients per provider hour

	Academic EDs (*n* = 80)	Non-Academic EDs (*n* = 454)	Matched Non-Academic EDs (*n* = 80)
Mean patients per hour of attending coverage			
Median (95% CI)	2.56 (2.47 to 2.78)	3.13 (3.00 to 3.22)	2.69 (2.51 to 2.95)
IQR (95% CI)	2.23 (1.93 to 2.43) – 2.99 (2.81 to 3.18)	2.61 (2.53 to 2.70) – 3.65 (3.53 to 3.79)	2.26 (2.07 to 2.44) – 3.33 (3.03 to 3.62)
Non-responders	4 (5.0%)	122 (27%)	18 (23%)
Mean patients per hour of attending + APP coverage			
Median (95% CI)	1.93 (1.80 to 2.16)	2.29 (2.26 to 2.36)	2.16 (2.04 to 2.31)
IQR (95% CI)	1.69 (1.51 to 1.78) – 2.38 (2.16 to 2.69)	2.06 (2.00 to 2.15) – 2.60 (2.53 to 2.66)	1.88 (1.76 to 1.99) – 2.54 (2.35 to 2.71)
Non-responders	4 (5.0%)	121 (27%)	18 (23%)
Mean patients per hour of attending + APP + resident coverage			
Median (95% CI)	0.88 (0.79 to 1.02)	1.85 (1.81 to 1.88)	1.80 (1.70 to 1.92)
IQR (95% CI)	0.74 (0.66 to 0.77) – 1.25 (1.04 to 1.48)	1.66 (1.64 to 1.70) – 2.08 (2.02 to 2.13)	1.58 (1.48 to 1.67) – 2.09 (1.96 to 2.21)
Non-responders	4 (5.0%)	122 (27%)	18 (23%)

*APP* Advanced practice provider, *CI* Confidence interval, *IQR* Interquartile range

### Secondary analysis

Observed differences between academic and non-academic EDs persisted in the secondary analysis when comparing only within the 1:1 matched sample. Results for the matched sample of non-academic EDs are shown in Tables 2, 3 and 4.

## Discussion

Our investigation revealed that academic and non-academic EDs differed in multiple aspects of clinical operations, exhibiting key differences in demographics, ED throughput, flow of admitted patients, staffing and resource utilization, the majority of which persisted in the 1:1 matched sample analysis. These observations in total are important because they suggest that academic and non-academic EDs are not relevant comparators for benchmarking of clinical performance measures.

Demographic factors drive ED operations, perhaps none more so than annual patient volume [15]. Academic EDs exhibited significantly higher patient volumes than their non-academic counterparts, even after exclusion of ED with fewer than 40,000 annual encounters. In fact, nearly two thirds of non-academic EDs cared for fewer than 60,000 patients per year, while three quarters of academic EDs treated more than 60,000. Furthermore, fewer than one-tenth of non-academic EDs saw more than 80,000 patients per year, while over a third of academic EDs did the same. Our findings indicate the potential for greater complexity in academic ED operations based solely on greater patient volume.

Injury and disease acuity and severity also intuitively drive complexity of ED operations [16]. The admission rate was higher in academic EDs indicating that, as a group, they likely care for more acute and complex patient populations. Another indirect measure of acuity, high CPT codes, did not differ between the two types of EDs in our study. However, CPT leveling can be influenced by documentation practices and may under-represent patients at the high end of the acuity spectrum [17]. A third surrogate marker of acuity in our study was patients arriving via EMS -- these patients were more likely to be admitted than patients arriving by other means in both cohorts. The hospitalization rate of patient arriving by EMS did not differ between the academic and non-academic EDs, indicating similar acuity and complexity of EMS patients between the groups (as opposed to suggesting population-level differences in access to non-emergency transportation). However, the proportion of patients arriving via EMS was higher among academic EDs, suggesting that these higher-acuity patients may be over-represented in academic settings. This difference was attenuated in the matched sample analysis, which was likely an artifact of our matching approach, accounting for ED admission rate and volume. If patients arriving via EMS were admitted at approximately equal rates among academic and non-academic EDs, on average, sites with a higher proportion of patients arriving via EMS were likely to have a higher admission rate. This probably represented higher acuity and complexity in the patient population, rather than operational differences. While unsurprising, this observation does illustrate the difficulty of selecting like comparator EDs between academic and non-academic cohorts.

Also potentially serving as an indirect marker of acuity and complexity, nearly all of the academic EDs in our study were designated as Level I or Level II American College of Surgeons Trauma Centers. Only one fifth of non-academic EDs held the same designation. While trauma designation does not directly indicate higher patient acuity, trauma level designations do carry specific requirements [18], suggesting that these EDs at least are prepared to care for patients of higher complexity. There are many examples of well-resourced non-academic trauma centers, of course, but the resources required to achieve higher level trauma designations are more commonly found in academic settings.

Finally, the academic cohort cared for a lower proportion of pediatric patients than the non-academic cohort, which stands to reason as many large academic centers have separate pediatric EDs, which were excluded from our analysis. This likely explains why this difference persisted in the matched sample analysis. On average, pediatric patients appear to be characterized by lower acuity and complexity given that they have lower ED triage acuity scores and lower admission rates [19]. Thus, some of our observed differences in admission rate between academic and non-academic EDs may also be driven by unbalanced dilution with pediatric patients in non-academic settings.

The interplay of the demographic factors discussed above plus other unmeasured factors such as potentially disparate tertiary referrals and how these factors contribute to acuity and complexity of ED patient populations remain unclear. The proportion of patients who were admitted for hospitalization was clearly higher in academic EDs in our primary analysis, although the differences were attenuated in our matched sample. In fact, in our primary analysis, the lowest quartile for admission rate for academic EDs was similar to that of the highest quartile of non-academic sites. While it is possible that local practice variation may have contributed in some part to these findings, we feel that the magnitude of the observed difference is a strong indicator that true acuity and complexity differences do exist between academic and non-academic EDs overall.

With regard to imaging resource utilization, our investigation was limited by differences in how the surveys queried their participants. Based on these differences, one would expect that imaging utilization would appear higher among non-academic EDs, given that they reported total tests per 100 patients, as opposed to the academic cohort, which reported the percentage of patients getting at least one study within an imaging modality category. A hypothetical example of a patient undergoing a head and cervical spine CT illustrates this discrepancy: the non-academic cohort would have counted this as two CT studies being performed, while the academic cohort would only have one occurrence of CT being counted. Nonetheless, we found that academic centers used MR imaging more frequently, even with this bias toward the non-academic cohort reporting more testing, although this observation extinguished in the matched sample analysis. The reasons for this difference are unclear, however potential explanations include differing patient populations, differing sub-specialist support, different availability of MRI scanners, and local cultural difference related to testing. The fact that matched non-academic EDs utilized MRI at a rate similar to academic EDs suggests that MRI use may be an effect of patient complexity and acuity, more than provider preference. We did not find differences within the other imaging modalities, with the exception of the matched sample analysis in which non-academic EDs had slightly higher x-ray utilization. It remains possible that academic centers may exhibit higher utilization in all imaging modalities with the signal having been masked by the limitation related to the query methods. Further research with standardized definitions is warranted to better describe imaging resource utilization differences across the two types of EDs.

Our study indicated that arrival-to-provider time was similar in academic and non-academic EDs; however, median LOS was longer in academic settings overall and for admitted and discharged patients. ED boarding appeared to contribute to longer LOS in academic EDs, since boarding time was over 33% longer for that cohort. Boarding appeared to be volume-dependent in the primary analysis, ranging from 70 minutes in the smallest volume EDs to 195 minutes in EDs that saw more than 120k patients. Since academic EDs tended to be larger, it is unclear whether differences in boarding among volume strata were truly a reflection of the size of the ED or its affiliated hospital (presumably larger hospitals would have larger EDs). While differences in boarding times were less pronounced in the matched cohort analysis, they remained significant nonetheless, suggesting that differences in hospital operations at academic centers may drive substantial differences in ED flow, despite similar ED patient acuity and volume. Better understanding of the causal factors for boarding is warranted, especially in academic centers which appear to be disproportionately affected. Other potential causes for our observed LOS differences may include: differing patient populations, differing sizes and complexity of the EDs, differing practice cultures related to extent of ED evaluations and interventions (i.e. these preferentially occur in the ED rather than the inpatient or outpatient setting after disposition), differing inpatient operations and differing consultant practices [13]. Finally, the educational and research missions of academic EDs also cannot be excluded as potential contributors to the throughput differences, although there exist a number of large academic EDs in our dataset with timeliness measures substantially better than the average non-academic ED. Thus, timeliness differences cannot be fully attributed to the academic mission alone.

Academic EDs also exhibited higher LBTC rates, a commonly cited indirect indicator of ED flow [10], even in the matched analysis. The EDBA survey did not report sub-categories of LBTC [10], so it remains unclear how the subcategories differed between the groups. Academic and non-academic EDs provided initial evaluation equally quickly, suggesting that leaving before being evaluated by a provider was likely similar for the two cohorts. If true, it suggests that patients in academic EDs may have left more frequently during their ED care after their initial evaluation. This may have been due to extended throughput times or potentially other unmeasured patient dissatisfiers.

PPH is another metric commonly used to measure operational efficiency. Analysis of attending coverage alone and attending plus advance practice provider (APP) coverage, revealed that mean PPH metrics were lower for academic programs in the primary analysis, but the differences extinguished in the matched analysis. When also considering resident coverage, the observed difference was not only more pronounced in the primary analysis, it also persisted in the matched analysis. The causes for the constellation of findings related to PPH are difficult to determine, but our results support that it is multi-factorial. It appeared that the teaching mission did affect PPH. On the surface, this may seem intuitive given that academic attending physicians teach while seeing patients, which will necessarily require time not committed to direct patient care, however a greater number of “extender” providers (residents) did not appear to compensate for this. We suspect that this is due at least in part to the requirement that attending physicians directly supervise resident care (i.e. personally evaluate each patient) while the level of supervision of APPs can be highly variable. In addition, the demands of student supervision, which were not measured in our analysis, also likely contributed to our findings. The observation that the matched analysis revealed no diffidence in PPH when considering attending and attending plus APP coverage provides evidence that the teaching mission is not the sole influence in the observed differences in PPH productivity. These other causes remain unclear, but they were likely similar to those discussed in the LOS discussion above.

A few limitations in our study methodology warrant consideration when interpreting the results. The two cohorts differed in their distributions across the volume sub-cohorts. ED flow variations are known to be associated with volume [10]. Lower volume EDs predominate the non-academic cohort, favoring more efficient ED operations. However, the slower ED throughput metrics do appear to be consistent within the volume sub-cohorts. It remains unclear if the differing distribution of ED volumes influenced the results. The two cohorts also differed in regional distributions, raising the potential for unmeasured regional practices having influenced the results. Our secondary, matched analysis attempted to mitigate some of these limitations, however such an approach is not perfect.

The selection of potential survey participants also differed between the two surveys. AAAEM/AACEM was distributed to all eligible academic programs, and EDBA was distributed to EDBA members and institutions that had participated in the past. This may have created selection bias, potentially favoring EDs with heightened interest in operational processes and improvement. The survey time periods differed by six months, but there was a six month overlap and both covered a full, continuous year of data. We believe it unlikely that this affected the outcomes. While the overall response rates were favorable for both surveys, there was variability in response rates for individual survey questions with some between-survey differences, especially in the utilization data. How this may have affected the results remains unclear.

Finally, our inclusion and exclusion methodology was designed to ensure greater specificity related to programs being academic versus non-academic. We believe it unlikely that we incorrectly excluded any true primary academic institutions from the AAAEM/AACEM database, however it is possible that we did exclude some true non-academic sites from the EDBA survey. This number is likely small, and it remains unclear how it may have affected the results. Nonetheless, we believe the methodology was sound in its goal of identifying true academic versus true non-academic sites.

Finally, it is prudent to emphasize that the differences in clinical measures observed between academic and non-academic EDs did not allow for conclusions related to causality. One may be tempted to presume that any metric in which the academic and non-academic centers performed differently was due simply to the academic mission itself. However, our results demonstrate clearly that the two cohorts differ even in the most basic demographic characteristics, which intuitively must contribute in some part to the observed results. Furthermore, other factors unmeasured in our investigation are certain to exist. Better understanding of the root causes of the observed differences between academic and non-academic EDs is warranted as it will likely provide knowledge with which to improve clinical operations in both types of EDs.

## Conclusions

Our investigation demonstrated important differences in demographic and operational performance measures between academic and non-academic EDs. These results suggest that the two groups may be inappropriate operational performance comparators, especially when considered in the aggregate without robust and careful matching. Causes for the differences between academic and non-academic EDs remain unclear, but one should not presume them necessarily or entirely to be due to the academic mission itself. Further investigation into causal factors is warranted as this may provide information to improve operations of both types of EDs.

## Supplementary information

**Additional file 1:** **Table S1.** AAAEM/AACEM ED Benchmarking Survey, Select Questions and Definitions. List of AAAEM/AACEM survey questions and associated definitions

**Additional file 2: Table S2.** EDBA Benchmarking Survey, Select Questions and Definitions. List of EDBA survey questions and associated definitions

## Data Availability

The datasets generated and/or analyzed during the current study are not publicly available due the fact that both datasets included clinical operations performance data that could be identified to individual institutions. Furthermore, part of the incentive for institutions to participating in the surveys was to receive summaries of the outcomes. Publically reporting the datasets would erode this incentive. The datasets are available from the corresponding author on reasonable request with agreement of non-disclosure and consent of AAAEM/AACEM and EDBA

## Abbreviations

AAAEM: Academy of Academic Administrators of Emergency Medicine
AACEM: Association of Academic Chairs of Emergency Medicine
ACGME: Accreditation Council for Graduate Medical Education
APP: Advanced practice provider
CI: Confidence interval
CPT: Current Procedural Terminology (a registered trademark of the American Medical Association)
CT: Computed tomography
ED: Emergency department
EDBA: Emergency Department Benchmarking Alliance
EM: Emergency medicine
EMS: Emergency medical services
IQR: Interquartile range
LBTC: Left Before Treatment Complete (LBTC)
LCME: Liaison Committee on Medical Education
LOS: Length of stay
MRI: Magnetic resonance imaging
PPH: Patients per provider-hour
US: Ultrasound
US: Unites States (US)
