# Supplementary material for: Clinical operations of academic versus non-academic emergency departments: a descriptive comparison of two large emergency department operations surveys
Source: BMC Emerg Med. 2019 Nov 21;19:72. doi: 10.1186/s12873-019-0285-7 (PMC6868754; doi:10.1186/s12873-019-0285-7)
Supplement: Supplementary file 1 — Additional file 1: Table S1. AAAEM/AACEM ED Benchmarking Survey, Select Questions and Definitions. List of AAAEM/AACEM survey questions and associated definitions [file 12873_2019_285_MOESM1_ESM.pdf]

Supplement Table 1. AAAEM/AACEM ED Benchmarking Survey, Select Questions and Definitions

Italics denotes calculated fields for comparison to EDBA values.

Question	Definition
What level trauma center are you?	Your institution's trauma center level designation (generally I or II).
Hospital Admissions (exclude deliveries/nursery)	Number of hospital-wide admissions, including those from the ED, but excluding deliveries/nursery and observation status at discharge
Hospital Observations (exclude ED-based observations)	Number of hospital-wide observations, including those that came from the ED, but excluding ED-based observations (these will be counted separately later)
Total Hospitalizations	*Sum of Hospital Admissions and Hospital Observations*
Total Number of Discharges (include discharged observation patients, transfers and walkouts)	Number of patients who were seen and discharged, including those placed in ED OBS (not admitted as an inpatient to your hospital). Please include transfers and walkouts.
Total Number of Admissions	Number of patients who were seen and admitted as a hospital inpatient.
Total Number of Hospital Observations (exclude ED-based observations)	Number of patients who were seen and sent to an inpatient bed for observation. Do not include ED-based observations.
Total ED Volume	*Sum of Total Discharges, Admissions, and Hospital Observations above.*
% ED Patients Admitted	*(Total Number of Admissions + Total Number of Hospital Observations) / Total ED Volume*
% Hospital Admissions Through the ED	*(Total Number of Admissions + Total Number of Hospital Observations) / (Hospital Admissions + Hospital Observations)*
Number of Pediatric Visits	Number of pediatric visits, as defined by your institution. Exclude those patients not in the dataset being used to complete this survey.
% Pediatric Visits	*Number of Pediatric Visits / Total ED Volume*
Number of EMS Arrivals (exclude intra-facility transports)	All EMS arrivals; air or ground (excluding intra-facility transports)
Number of EMS Arrivals Hospitalized: After ED Evaluation	All EMS arrivals that we admitted or sent to an inpatient bed for observation after receiving ED evaluation/care
% EMS Arrivals	*Number of EMS Arrivals / Total ED Volume*
% EMS Admitted	*Number of EMS Arrivals Hospitalized / Number of EMS Arrivals*
Total Number of Patients that (LWBS) Left Without Being Seen by a Provider	Patients who left after registration, but before being screened by a QMP in triage (if present) or seen by a provider. LWOT/CMS reported value.
Total Number of Patients that were Screened and Left	Patients who registered and were screened by a QMP provider, but left before management/ treatment was fully started in a bed or equivalent space
Total Number of Patients that Left Against Medical Advice (AMA) or Eloped	Patients who left against the advice of the medical provider or eloped from a treatment room without signing an AMA form. This is intended to represent patients being actively managed/treated, not just screened by a QMP.
% LBTC (Left Before Treatment Complete)	*(Total LWBS + Total Screened and Left + Total AMA or Eloped) / Total ED Volume*
Total Number of Acute Treatment Spaces	

(exclude hallway beds)	
Total Number of Fast Track/Intake/Front-End Treatment Spaces	
Total Number of Dedicated Observation Medicine Treatment Spaces	
Total Number of Dedicated Behavioral Health/Psychiatry Spaces	
Total Treatment Spaces	*Acute Treatment Spaces + Fast Track/Intake/Front-End Treatment Spaces + Dedication Observation Medicine Treatment Spaces + Dedicated Behavioral Health / Psychiatry Treatment Spaces*
Visits Per Treatment Space	*Total ED Volume / Total Treatment Spaces*
What is the gross square footage of the ED?	
Median Length of Stay for All Patients in Hours	Median time from arrival to left department for patients of all dispositions, excluding psych and walkouts (LWBS, SAL, AMA, Eloped).
Median Length of Stay for Admitted/Hosp Obs Patients in Hours	Median time from arrival to left department for admitted and hospital-based observation patients only, excluding ED observations and psych.
Median Length of Stay for Discharged Patients in Hours	Median time from arrival to left department for discharged patients only, excluding ED observations, psych and walkouts (LWBS, SAL, AMA, Eloped).
Median Time from Arrival to Seen by Provider for All Patients (Door to Doc)	Median time from arrival to being seen by a provider (qualified to do the medical screening exam).
Median Time from Decision to Hospital Unit (boarding) for Admitted/Hosp Obs Patients	Median time from the admit or inpatient observation decision/order to when the patient left the ED for their inpatient bed. CMS definition is from the ED record only, ED provider decision, regardless of before or after an accepting consult or conversation. Exclude psych patients.
Professional Fee Billing: Total Number of Level 4 (99284) Patients	CPT 99284
Professional Fee Billing: Total Number of Level 5 (99285) Patients	CPT 99285
Professional Fee Billing: Total Number of Critical Care (99291) Patients	CPT 99291
% CPT High Acuity	*(Professional Fee Level 4 + Level 5 + Critical Care 99291) / (Professional Fee Level 1 + Level 2 + Level 3 + Level 4 + Level 5 + Critical Care 99291)*
Total number of visits that had at least one CT ordered by an ED provider	Total number of patient visits (not number ordered) that had at least one CT ordered by an ED provider.
% Patients Receiving a CT	*Total visits with at least one CT ordered / Total ED Volume*
Total number of visits that had at least one MRI ordered by an ED provider	Total number of patient visits (not number ordered) that had at least one MRI ordered by an ED provider.
% Patients Receiving an MRI	*Total visits with at least one MRI ordered / Total ED Volume*
Total number of visits that had at least one plain film ordered by an ED provider	Total number of patient visits (not number ordered) that had at least one plain film ordered by an ED provider.
% Patients Receiving an X-Ray	*Total visits with at least one plain film ordered / Total ED Volume*

Total number of visits that had at least one ultrasound ordered by an ED provider AND performed by Radiology	Total number of patient visits (not number ordered) that had at least one ultrasound ordered by an ED provider and performed by Radiology.
% Patients Receiving an US	*Total visits with at least one US ordered / Total ED Volume*
Emergency Department Physician Hours (per day, exclude charting after assigned shift)	Actual hours PER DAY of physician direct patient care in the main ED, excludes charting after assigned shift Per Day
Observation Unit Physician Hours (per day)	Actual hours PER DAY of physician direct patient care in the observation unit, if unit exists, exclusive of charting after assigned shift Per Day
Intake/Low Acuity/Fast Track Physician Hours (per day)	Actual hours PER DAY of physician direct patient care in Intake/Low Acuity/Fast Track unit, if unit exists, exclusive of charting after assigned shift Per Day
Total Daily Physician Hours	*ED Physician Hours + Obs Unit Physician Hours + Intake/Fast Track/Low-Acuity Physician Hours*
Emergency Department APP Hours (per day)	Actual hours PER DAY of APP (NPs, PAs, etc.) direct patient care in the main ED exclusive of charting
Observation Unit APP Hours (per day)	Actual hours PER DAY of APP direct patient care in the obs unit, if unit exists, exclusive of charting
Intake/Low Acuity/Fast Track APP Hours (per day)	Actual hours PER DAY of APP direct patient care in Intake/Low Acuity/Fast Track unit, if unit exists, exclusive of charting
Total Daily APP Hours	*ED APP Hours + Obs Unit APP Hours + Intake/Fast Track/Low-Acuity APP Hours*
Emergency Department Resident Hours (per day)	Actual hours PER DAY of resident direct patient care (including off service residents) in the main ED, exclusive of charting
Observation Unit Resident Hours (per day)	Actual hours PER DAY of resident direct patient care in the observation unit, if unit exists, exclusive of charting
Intake/Low Acuity/Fast Track Resident Hours (per day)	Actual hours PER DAY of resident direct patient care in Intake/Low Acuity/Fast Track unit, if unit exists, exclusive of charting
Total Daily Resident Hours	*ED Resident Hours + Obs Unit Resident Hours + Intake/Fast Track/Low-Acuity Resident Hours*
Total Daily Provider Hours	*Total Daily Physician Hours + Total Daily APP Hours + Total Daily Resident Hours*
Patients Per Attending Hour	*(Total ED Volume / 365) / Total Daily Physician Hours*
Physician + APP Ratio	*(Total ED Volume / 365) / (Total Daily Physician Hours + Total Daily APP Hours/2)*
Do you utilize scribes?	
Average total number of scribe hours per day	Total number of scribe hours per day. For example, if you have three 8-hour scribes shifts each day, put 24.
