# Supplementary material for: Clinical operations of academic versus non-academic emergency departments: a descriptive comparison of two large emergency department operations surveys
Source: BMC Emerg Med. 2019 Nov 21;19:72. doi: 10.1186/s12873-019-0285-7 (PMC6868754; doi:10.1186/s12873-019-0285-7)
Supplement: Supplementary file 2 — Additional file 2: Table S2. EDBA Benchmarking Survey, Select Questions and Definitions. List of EDBA survey questions and associated definitions [file 12873_2019_285_MOESM2_ESM.pdf]

Supplement Table 2. EDBA Benchmarking Survey, Select Questions and Definitions

Italics denotes calculated fields for comparison to AAAEM/AACEM values.

Question	Definition
Is this a Trauma Center Level I, II, III, or IV or no designation?	
Do you participate in training Emergency Medicine residents?	
What were the total ED Visits for 2017?	
What % of ED patients were admitted?	Includes BOTH full and observation admissions
What % of TOTAL hospital admissions come through the ED?	Includes full and observation admissions
% of patients under age 2?	
% of patients between 2 and 18 years of age?	
% Pediatric Visits	% of patients under age 2 + % of patients between 2 and 18 years of age
What % of patients arrived by EMS?	
Of patients arriving by EMS, what % admitted?	
What % patients Left Before Treatment was Complete (LBTC)?	Includes all patients who left before or after triage, eloped, left AMA, or any other descriptor used to identify patients who leave before treatment is complete.
How many patient care spaces in the ED (or ED beds)?	
Visits Per Treatment Space	*Total ED Visits / Patient Care Spaces*
What is gross square footage of ED?	
MEDIAN Length of Stay for ALL patients?	
MEDIAN Total Length of Stay for Admitted Patients?	
MEDIAN Length of Stay for Treat and Release Patients?	
MEDIAN time for patients from Door to “Bed”?	
MEDIAN time for patients from “Bed” to “Doctor Sees Patient”?	
MEDIAN Admit decision to departure time (CMS Measure)?	
What % High Acuity	Physician CPT code level 4 + 5 + critical care
Studies Used in ED: CT Scans	Number of these procedures performed per hundred patients seen
Studies Used in ED: MRI Images	Number of these procedures performed per hundred patients seen
Studies Used in ED: Ultrasounds	Number of these procedures performed per hundred patients seen
Studies Used in ED: Simple Xrays	Number of these procedures performed per hundred patients seen
The Number of Clinical Staff Hours in an Average Clinical Day: MD	
The Number of Clinical Staff Hours in an Average Clinical Day: APP	
The Number of Clinical Staff Hours in an Average Clinical Day: Resident	
Total Daily Provider Hours	*Total MD Staffed Clinical Hours + Total APP Staffed Clinical Hours + Total Resident Staffed*

	Clinical Hours
Patients Per Attending Hour	*(Total ED Visits / 365) / Total MD Staffed Clinical Hours*
Physician + APP Ratio	*(Total ED Visits / 365) / (Total MD Staffed Clinical Hours + Total APP Staffed Clinical Hours/2)*
The Number of Clinical Staff Hours in an Average Clinical Day: Scribes	
